# Blood–brain barrier permeability of normal‐appearing white matter in patients with vestibular schwannoma: A new hybrid approach for analysis of *T*_1_‐W DCE‐MRI

**DOI:** 10.1002/jmri.25573

**Published:** 2017-01-24

**Authors:** Ka‐Loh Li, Xiaoping Zhu, Sha Zhao, Alan Jackson

**Affiliations:** ^1^ Division of Informatics, Imaging and Data SciencesUniversity of ManchesterManchesterUK; ^2^ CRUK and EPSRC Cancer Imaging Centre in Cambridge and ManchesterManchesterUK

**Keywords:** DCE‐MRI, blood‐brain barrier permeability, normal appearing white matter, first‐pass model, Patlak plot, neurofibromatosis type 2

## Abstract

**Purpose:**

To develop and assess a “hybrid” method that combines a first‐pass analytical approach and the Patlak plot (PP) to improve assessment of low blood–brain barrier permeability from dynamic contrast‐enhanced (DCE) magnetic resonance imaging (MRI) data.

**Materials and Methods:**

Seven patients with vestibular schwannoma were enrolled. *T*_1_‐W DCE imaging was acquired on a 1.5T scanner. Normal‐appearing white matter (NAWM) was divided into four regions of interest (ROIs) based on the magnitude of changes in longitudinal relaxation rate (ΔR1) after gadolinium administration. Kinetic analysis of ROI‐averaged contrast agent concentration curves was performed using both the conventional PP and the hybrid method. Computer simulated uptake curves that resemble those from NAWM were analyzed with both methods. Percent deviations (PD) of the “measured” values from the “true” values were calculated to evaluate accuracy and precision of the two methods.

**Results:**

The simulation showed that, at a noise level of 4% (a noise level similar to the in vivo data) and using a signal intensity (SI) averaging scheme, the new hybrid method achieved a PD of 0.9 ± 2.7% for *v*_p_, and a PD of –5.4 ± 5.9% for *K* ^trans^. In comparison, the PP method obtained a PD of 3.6 ± 11.3% for *v*_p_, and –8.3 ± 12.8% for *K* ^trans^. One‐way analyses of variance (ANOVAs) showed significant variations from the four WM regions (*P* < 10^−15^ for ΔR1; *P* < 10^−6^ for *K* ^trans^; *P* < 10^−4^ for *v*_p_).

**Conclusion:**

Both computer simulation and in vivo studies demonstrate improved reliability in *v*_p_ and *K* ^trans^ estimates with the hybrid method.

**Level of Evidence:** 3

**Technical Efficacy:** Stage 1

J. MAGN. RESON. IMAGING 2017;46:79–93

Quantification of low blood–brain barrier (BBB) permeability from dynamic contrast‐enhanced (DCE) magnetic resonance imaging (MRI) is associated with a number of technical challenges. The analysis must be based on pharmacokinetic analysis of time course data, which requires accurate separation of the contribution of several variables. This can be problematic where the signal‐to‐noise ratio and temporal resolution are low, as is the case where BBB permeability is low, resulting in inappropriate covariance between parameters derived from curve‐fitting approaches. A common approach to these problems is the use of the Patlak method,^1^, ^2^, ^3^, ^4^, ^5^, ^6^ where the slope in the Patlak plot (PP) represents the unidirectional influx constant (*K* ^trans^), and the y‐intercept represents the fractional plasma volume (*v*_p_). In a previous study, Ewing et al found higher *v*_p_ values from Patlak analysis compared to those estimated by integrating the area under the contrast concentration time course over the first‐pass (FP) transit period of the contrast agent (CA) bolus.^3^ This might reflect the weakness of the Patlak method, which depends on linear regression analysis, and is more prone to nonuniform (distorted) noise. In addition, the model does not allow for backflux of CA from the tumor into the plasma, which will be of particular importance if an inappropriate time interval is chosen for analysis.

In this study we propose a new “hybrid” method that combines a first‐pass analytical approach^7^, ^8^ with the Patlak plot (PP) to improve assessment of BBB permeability. The PP model describes a unidirectional two‐compartment system and uses linear regression analysis to estimate *K* ^trans^ and *v*_p_. The FP method^7^, ^8^ performs an automatic decomposition of the first‐pass CA concentration curve into intravascular and interstitial components to allow simultaneous mapping *K* ^trans^ and *v*_p_. A leakage‐corrected estimate of *v*_p_ is obtained by integrating the area under the intravascular CA concentration curve over the first‐pass of the bolus. When combined with high temporal resolution DCE data, the FP method provides accurate and robust measurements of *v*_p_.^2^, ^9^

The aim of this study was to develop an easy‐to‐use and reliable approach for detecting subtle BBB permeability in normal‐appearing white matter (NAWM) and to apply the proposed techniques in patients with type II neurofibromatosis‐associated vestibular schwannoma (VS). We hypothesize that a hybrid approach combining the benefits of the FP and PP analytical approaches will improve the accuracy of parameter estimates in tissues with low permeability.

## Theory

### Patlak Plot

The Patlak model describes a highly perfused two‐compartment tissue assuming unidirectional transport from the plasma into the extravascular extracellular space (EES). The CA concentration in tissue is given by:

(1) $$C_{t}(t)\;=\;v_{p}C_{p}(t)+K^{trans}\;\int_{0}^{t}C_{p}(\tau )d\tau ,$$

where *C*_p_(*t*) is the plasma CA concentration time course curve, *v*_p_ is the fractional plasma volume, and *K* ^*trans*^ is the volume transfer constant between blood plasma and the leakage space. Dividing both sides of Eq. [1] with *C*_p_(*t*), one obtains:

(2) $$\frac{C_{t}(t)}{C_{p}(t)}=v_{p}+K^{trans}\frac{\int_{0}^{t}C_{p}(\tau )d\tau }{C_{p}(t)}.$$

**Figure 1:**
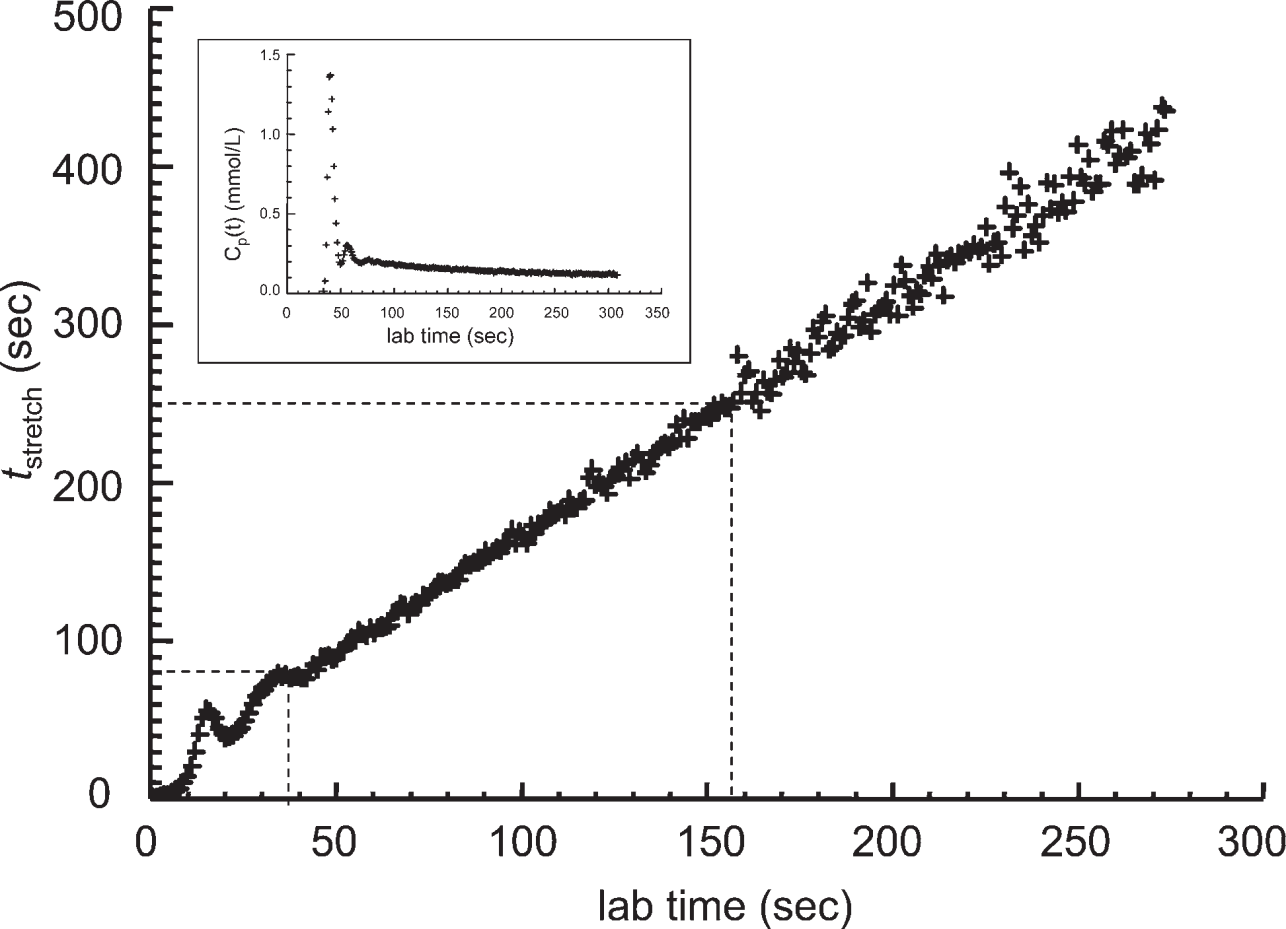
Relationship between *t*_stretch_, calculated as $\left(\;\int_{0}^{t}C_{p}(\tau )d\tau )/C_{p}(t\right)$, and the lab time. The panel in the upper‐left corner of the graph shows the *C*_*p*_(*t*) used in the simulation.

Equation [2] expresses the PP, where the slope represents *K* ^trans^ and the intercept represents *v*_p_. The term on the left side of the equation, *C*_*t*_(*t*)/*C*_*p*_(*t*), represents the volume of distribution (*v*_d_) of the CA in brain tissue at the time of sampling, *t*.^10^ If the BBB is intact, then *v*_d_ should be equivalent to *v*_p_. If leakage occurs, then *v*_d_ becomes larger than the *v*_p_, as accessible extravascular compartments are included.^2^ The abscissa has the units of time, but this is not laboratory time. It is concentration‐stretched time and will be referred to hereafter as *t*_stretch_.^3^ Figure 1 shows the relationship between the lab time and *t*_stretch_ calculated using a *C*_*p*_(*t*) measured from low CA dose, high temporal resolution (LDHT) imaging in a patient with VS.^11^ It can be seen that a *t*_stretch_ interval 80–250 seconds^2^ corresponds to a lab time interval 37–157 seconds. The relationship between *t*_stretch_ and lab time is approximately linear beyond a lab time of ∼37 seconds, but fluctuates within the first‐pass and recirculation phase of the CA bolus. In most studies permeability is evaluated during the steady‐state component of the plot, ignoring the initial bolus circulation. An additional problem is that experimental errors are distorted when a nonlinear model is transformed to a linear one.^12^ How these distorted errors and the initial timepoints affect the Patlak fitting will be investigated in the current study.

### FP Model for Simultaneously Deriving v_p_ and K^trans^ (FP_simul_)

The FP method^7^, ^8^ assumes that backflow during the first‐pass transit period of the bolus is negligible, and uses an iterative approach^8^ to separate the intravascular and interstitial components of the tissue CA concentration curve, which are then used for *v*_p_ and *K* ^trans^ estimation, respectively (see details in Appendix A).

### New Hybrid Method

The new hybrid method contains three parts:

1. a conventional PP for simultaneously deriving *K* ^trans^ and *v*_p_;
2. a modified FP analysis with a known *K* ^trans^ for deriving *v*_p_ only;
3. a modified PP analysis with a known *v*_p_ for deriving *K* ^trans^ only.

**Figure 2:**
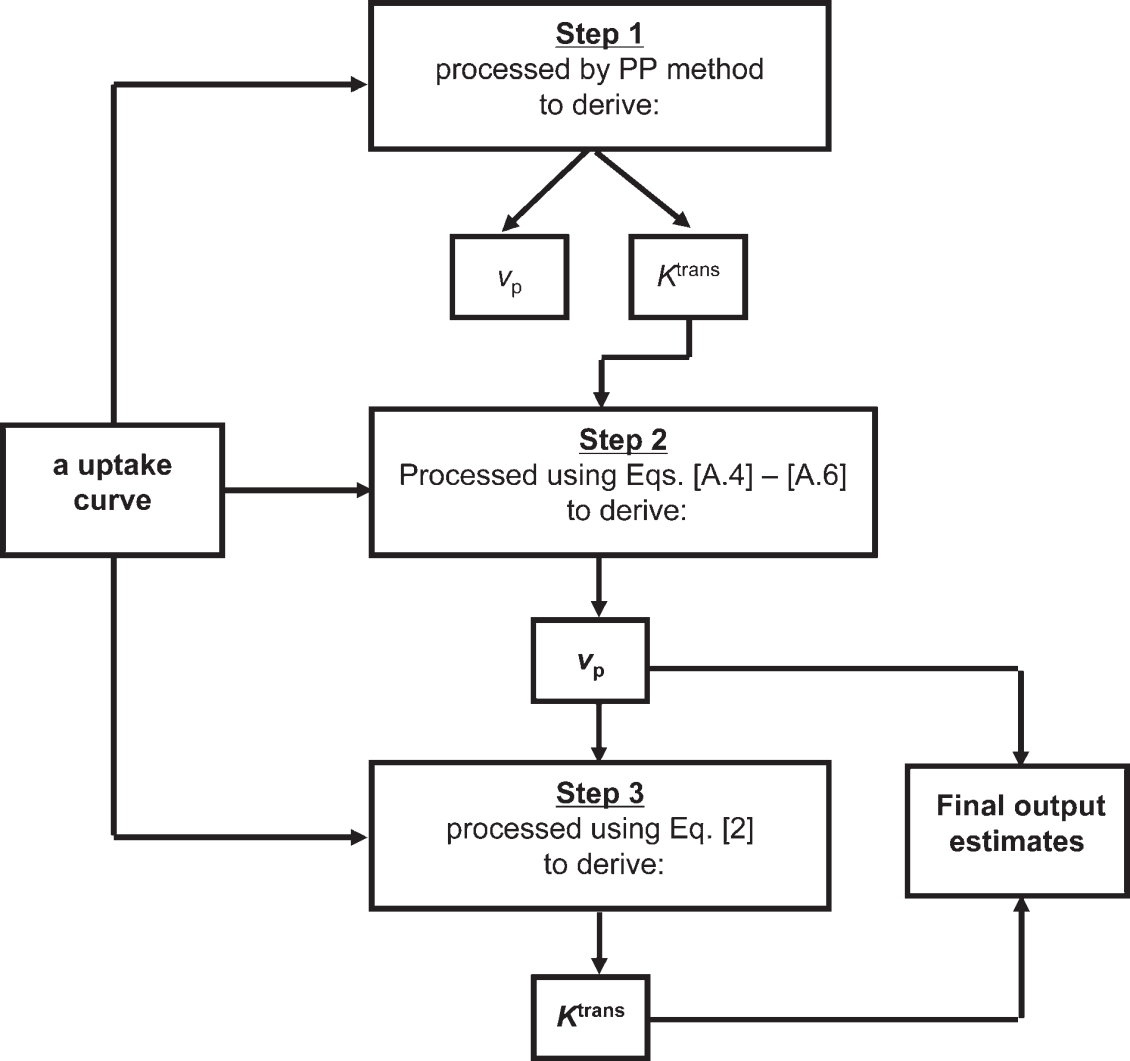
Flow chart of the FP‐PP hybrid method.

A flow chart of the new hybrid method is presented in Fig. 2. The analysis is performed in three steps:

1 Step 1: CA time course curves are fitted using the conventional PP to derive an initial estimate of *K* ^trans^ and *v*_p_ values, with a *t*_stretch_ interval of 85–250 sec used in the fitting;
2 Step 2: The estimate of *K* ^trans^ from Step 1 is used into Eq. [A.4] (Appendix A) as a known value and *v*_p_ is then calculated using Eqs. [A.5] and [A.6] (Appendix A);
3 Step 3: CA time course curves are then refitted with Eq. [2] to obtain a refined *K* ^trans^ estimate. During the fitting, the *v*_p_ in Eq. [2] is fixed using the value obtained in Step 2, leaving *K* ^trans^ as the only free‐fitting parameter.

The estimates of *v*_p_ from Step 2, and *K* ^trans^ from Step 3 are the final output estimates from the analysis.

The conventional PP performed at Step 1 provides an adequate estimate of *K* ^trans^ for use as the initial value^3^ in the modified FP method enabling subsequent calculation of *v*_p_, corrected for leakage. In low permeability tissues such as NAWM the intravascular CA concentration is very high compared to the amount of transendothelial leakage. This means that errors in *K* ^trans^ estimation from Step 1 will have only very small effects on the calculation of the integral of the intravascular CA time course curve in Step 2. Consequently, the modified FP approach becomes less sensitive to covariance errors that result from simultaneous measurement of *K* ^trans^ and *v*_p_. The resulting improved estimate of *v*_p_ is then used in the modified PP method, leaving *K* ^trans^ as the only free‐fitting parameter. The stepwise estimation of *K* ^trans^ and *v*_p_ may be expected to improve measurement accuracy and enable more reliable estimation in tissue with very low levels of endothelial permeability.

## MATERIALS AND METHODS

### Patients

Seven patients with type 2 neurofibromatosis (NF2), with a total of 20 tumors (14 VS and 6 meningiomas), were recruited into the study. DCE‐MRI studies were performed in the absence of any treatment. All subjects gave informed consent and the Local Research Ethics Committee approved the study (reference number O2‐051).

### MRI

Patients were imaged on a 1.5T whole body scanner (Philips Achieva, Philips Medical Systems, Best, Netherlands) using an 8‐channel head coil. LDHT DCE‐MRI data were collected as part of the dual temporal resolution technique, ICR‐DICE, as described previously.^11^ Prior to the LDHT DCE series, four consecutive 3D axial fast gradient recalled echo acquisitions (GRE) with variable flip angle (VFA; α = 2°, 8°, 15°, and 20°) were performed for native longitudinal relaxation rate (R1_N_ = 1/T1_N_) mapping. The fourth sequence was then repeated (*n* = 300) to produce a high temporal resolution (Δ*t* = 1.03 sec) *T*_1_‐W dynamic dataset with a low dose of CA (gadoterate meglumine; Dotarem, Geurbet, Roissy, France) given following the 30^th^ dynamic scan. Contrast agent (a fixed volume of 3 ml, ∼0.02 mmol/kg depending on body weight) was administered by power injector as an intravenous bolus at a rate of 3 ml/s, followed by a chaser of 20 ml of 0.9% saline administered at the same rate. Two sets of high spatial resolution *T*_1_‐W 3D images with the voxel size of 1 × 1 × 1 mm, ie, isotropic voxels, were acquired before (*T*_1_‐weighted [W] without contrast) and after the DCE MRI (*T*_1_‐W + contrast).

### Image Processing

Prior to kinetic analysis of the CA concentration curves observed in NAWM, we performed pixel‐by‐pixel mapping of postinjection changes in longitudinal relaxation rate (ΔR1) to show CA distribution due to BBB leakage in the brain tissue. A four‐color scheme was applied to the WM‐segmented ΔR1 maps, which divided the WM into four ROIs based on the magnitude of ΔR1. Kinetic analysis of ROI‐averaged CA concentration curves was then performed using both the conventional PP and the hybrid method. Computer simulations were performed to generate CA concentration curves that resemble the in vivo CA curves to evaluate accuracy (bias) and precision (standard deviation) of the new hybrid method.

#### Measuring Plasma CA Concentration Time Course Curve (*C*_p_(t))

The plasma CA concentration time course curve (*C*_p_(*t*)) was measured from the superior sagittal sinus (SSS) in patient data as shown in Fig. 1. A semiautomatic extraction technique was used in the *C*_p_(*t*) measurement (see Appendix B for details).

#### Calculation of R1_N_ and M_0_

Maps of M_0_ and R1_N_ were calculated by fitting the signal intensities from VFA images using a nonlinear least squares method (see details in Appendix C).

#### Pixel‐by‐Pixel Calculation of CA‐Induced R1 Changes

To reduce the influence of Rician noise, the average of the signal intensity (SI) in the last 10 dynamic frames (around 4.5 min postinjection) was used to calculate the postinjection tissue R1 (R1_post_). Calculation of R1_post_ based on R1_N_, mean preinjection signal intensity (SI_pre_), and postinjection signal intensity (SI_post_), with a subtraction method (SI_post_ – SI_pre_), is described in Appendix D.

Maps of ΔR1 ( = R1_post_ – R1_N_) were generated. To view the spatial distribution of the residual CA in brain tissues, WM‐segmented ΔR1 maps were displayed in four colors (blue, green, red, and yellow) based on the magnitude of ΔR1, and associated *v*_p_ values. The criteria setting for the color‐coding (blue: ΔR1 < 0 or *v*_p_ < 0.01; green: 0 < ΔR1 < 0.012 and *v*_p_ > 0.01; red: 0.012 ≤ ΔR1 < 0.025 and *v*_p_ > 0.01; and yellow: ΔR1 ≥ 0.025 and *v*_p_ > 0.01; *v*_p_ was estimated by integrating the area under the first‐pass CA concentration curves without leakage correction) was based on in vivo observation from a patient with NF2, and will be explained in more detail in the Results section below.

#### Segmentation of Gray and White Matter

SPM2^13^ was used for 1) spatial alignment between R1_N_ VFA, DCE‐MRI, and 3D *T*_1_‐W isotropic images, and 2) segmentation of the MRI data into GM, WM, and CSF. The probability maps of GM, WM, and CSF segmented from the *T*_1_‐W isotropic images were realigned and resliced to the space of the 3D individual frames of the DCE‐MRI, as well as the 3D R1_N_ and 3D pharmacokinetic parametric images, ie, *K* ^trans^ and *v*_p_. WM masks were generated from the WM probability maps by including only voxels with a probability greater than 0.95 and were used for the subsequent quantitative analysis.

#### Quantitation of Tissue BBB Permeability With the New Hybrid Method

ROI‐averaged (SI) time course data were used to test the new hybrid method. Each ROI was made as a collection of all the pixels with same color in a WM‐segmented image. Both conventional Patlak and the new hybrid methods were applied and compared. In addition, three *t*_stretch_ intervals (85–250 sec, 85–300 sec, and 0–250 sec) were used in the fitting to evaluate how much the specific time intervals could affect the results.

### Computer Simulation to Evaluate the New Hybrid Method

Based on the results from the in vivo data analysis, *K* ^trans^ = 0.0074 min^−1^ and *v*_p_ = 0.024 were chosen as “true” values to synthesize tissue CA concentration curves, which resemble the in vivo CA uptake curve in the yellow‐coded region. The *C*_*p*_(*t*) shown in Fig. 1 was used in the CA concentration curve simulation and fitting.

To investigate the effects on parameter estimation of the assumption that no backflux of CA occurs, zero noise tissue uptake curves were synthesized with the modified Tofts model^14^, ^15^ (assuming the fractional volume of the extravascular extracellular space, *v*_e_ = 0.20) and the unidirectional two‐compartment model, respectively. The modified Tofts model with no backflux corresponds to the unidirectional two‐compartment model expressed as Eq. [1]. Fitting errors due to ignoring backflux when using the PP and the new hybrid method were compared.

To investigate the effects of noise on parameter estimation, CA concentration curves were simulated with the modified Tofts model and converted into an SI‐time curve based on the in vivo mean baseline SI (470; 33 baseline frames); the precontrast *T*_1_ relaxation time (*T*_10_), and a literature value of longitudinal relaxivity (4.39 mM^−1^ sec^−1^).^16^ The generated SI‐time courses were sampled with a temporal resolution of 1.03 seconds. Rician white noise with noise level (ie, standard deviation / mean baseline signal) of 1%, 2%, 3%, 4%, and 5%, respectively, was added to the simulated SI‐time curves. *K* ^trans^ and *v*_p_ were calculated using the synthetic datasets to produce the so‐called “measured” values. Percentage deviations (PD) of the “measured” values from the “true” values were calculated as: PD = (measured – true)/true. Both the conventional PP and the new hybrid method were used for kinetic analysis. A total of 20,000 repetitions were performed for each method to produce mean and standard deviation (SD) of PD for each parameter estimates.

To investigate the effects of averaging SI curves on parameter estimation while using the two kinetic analysis methods, 100 individual SI‐time curves, simulated as described above, were averaged to resemble the ROI‐averaged SI curves observed from the in vivo WM yellow‐coded region. 200 repetitions were performed for each method to produce mean and SD of PD for each parameter estimates.

A *t*_stretch_ interval 85–250 seconds was used in the above Monte Carlo simulations. To investigate the effects of including the initial timepoints in the Patlak fitting, we repeated the above simulations using a *t*_stretch_ interval 0–250 seconds instead. We also repeated the simulations setting *K* ^trans^ at 0.004, 0.008, 0.012, 0.016, 0.020, 0.025, 0.030, 0.035 min^−1^, respectively (other parameters were fixed at *v*_e_ = 0.20, *v*_p_ = 0.024, and noise level of 4%), to identify what level of back‐diffusion (*k*_ep_ = *K* ^trans^/*v*_e_) will cause the new hybrid method to fail, as indicated by high absolute value of PD mean or high SD of PD.

### Further In Vivo Study

#### Correlation Between ΔR1 and *K* ^trans^ in WM

Mean ΔR1 of each of the four color‐coded WM regions were calculated for each slice in the ΔR1 image volumes. The unidirectional influx constant *K* ^trans^ and *v*_p_ were derived from the corresponding regional uptake curves using the new hybrid method. The whole *t*_stretch_ interval (from 0 to ∼460 sec) was used in the fitting, which was in agreement with the lab time interval used for calculation of ΔR1. Using longer *t*_stretch_ interval also benefits the measurement of very low BBB leakage (the red and green‐coded WM regions). Linear regression analysis was performed to evaluate the relationship of ΔR1 and the unidirectional influx constant *K* ^trans^.

#### Comparison of the Four Color‐Coded Regions in WM

Mean ΔR1, *K* ^trans^ and *v*_p_ measured in each of the four color‐coded WM regions were compared. Data are expressed as mean ± SD for the seven patients. Statistical tests were performed with 95% confidence intervals, using one‐way analysis of variance (ANOVA) to assess variations of the parameters in relation to the four color‐coded WM regions. Post‐hoc Tukey's honestly significant difference (HSD) test was performed to determine statistically significant differences among the mean parameter values for each pair of the WM regions.

#### Robust Stability Against Variation in the *t*_stretch_ Interval

For assessing the degree of variation in *K* ^trans^ and *v*_p_ while using various *t*_stretch_ intervals in the kinetic analysis, the coefficient of variation (CoV), defined as the ratio of the standard deviation to the mean, was computed. Robust stability against variation in the *t*_stretch_ interval was compared between the two kinetic methods.

### Statistical Analysis

The accuracy and precision of the two methods were compared by computer simulation of the mean and SD of percentage deviations for *K* ^trans^ and *v*_p_ estimates assuming the existence of backflux of CA and at varying levels of Rician noise. Stability of the two methods to variation in the *t*_stretch_ interval was evaluated by computing the CoV in *K* ^trans^ and *v*_p_ estimates derived from the in vivo data using a range of *t*_stretch_ intervals. Linear regression analysis was performed to assess the relationship between CA‐induced R1 changes and the unidirectional influx constant *K* ^trans^ in WM. Multiple comparisons of the regional means of ΔR1, *K* ^trans^, and *v*_p_ in the four color‐coded WM regions were performed using one‐way ANOVA, followed by all pairwise comparisons using Tukey's HSD test (alpha = 0.05). *P* < 0.05 was considered statistically significant.

## Results

### CA‐Induced R1 Changes in WM

**Figure 3:**
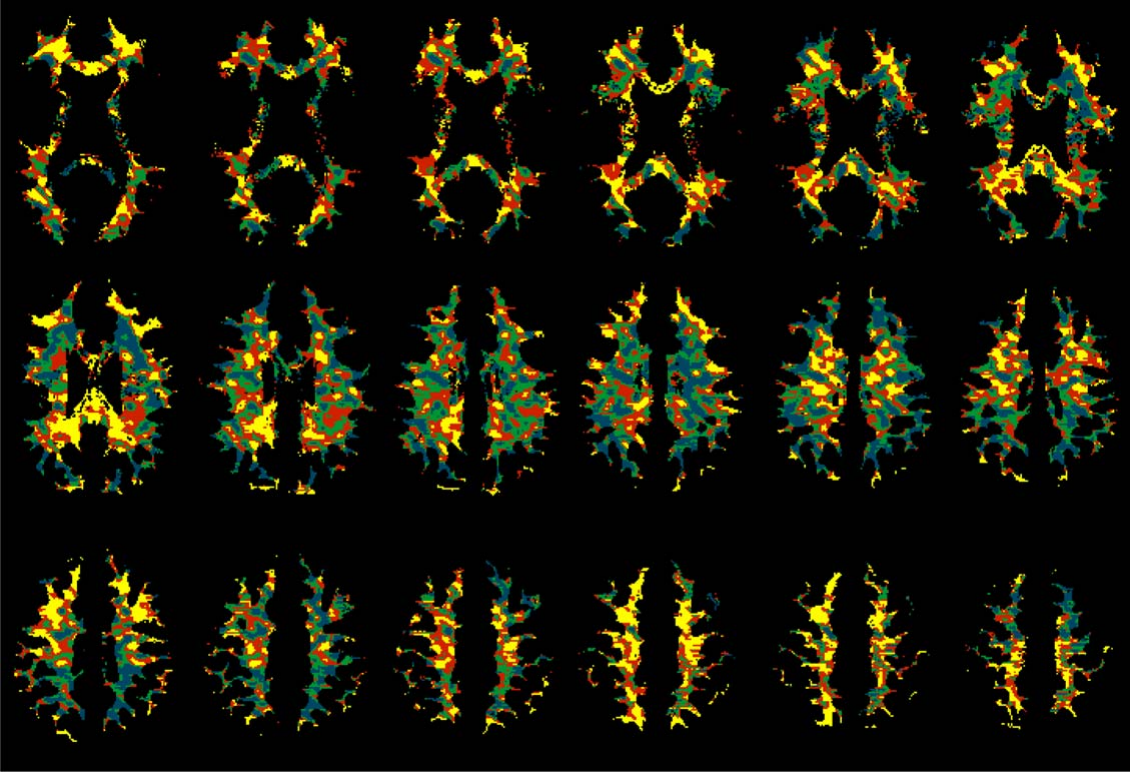
Regional variation in the distribution of residual contrast agent (CA) in white matter (WM) as shown with WM‐segmented ΔR1 maps (blue: ΔR1 < 0 or *v*_p_ < 0.01; green: 0 < ΔR1 < 0.012 and *v*_p_ > 0.01; red: 0.012 ≤ ΔR1 < 0.025 and *v*_p_ > 0.01; and yellow: ΔR1 ≥ 0.025 and *v*_p_ > 0.01; *v*_p_ was estimated by integrating the area under the first‐pass CA concentration curves without leakage correction).

Figure 3 shows maps of ΔR1 in WM measured around 4.5 minutes post‐CA injection in a patient with NF2 who had bilateral VSs and a frontal meningioma. The maps in Fig. 3 covers slices 37–54, whereas the right VS occurred in slices 14–24, the left VS in slices 15–17, and the meningioma in slices 57–61. From the ΔR1 maps it can be seen that the residual CA is not homogeneously distributed in WM.

It was found that about one‐fifth of the segmented WM voxels showed a negative ΔR1 value, and the WM voxels with negative ΔR1 tended to show low *v*_p_ estimated with the FP technique. In the above patient, WM voxels with negative ΔR1 had *v*_p_ 0.0116 ± 0.0107, while those with positive ΔR1 had *v*_p_ 0.0216 ± 0.0150; WM voxels with *v*_p_ < 0.01 had ΔR1 0.0028 ± 0.0141, while those with *v*_p_ > 0.01 had ΔR1 0.0161 ± 0.0163. In Fig. 3, the blue is the area of negative ΔR1. To reflect the low *v*_p_ attribute of the blue‐coded region, we included all WM voxels with *v*_p_ < 0.01 into the blue‐coded region.

**Figure 4:**
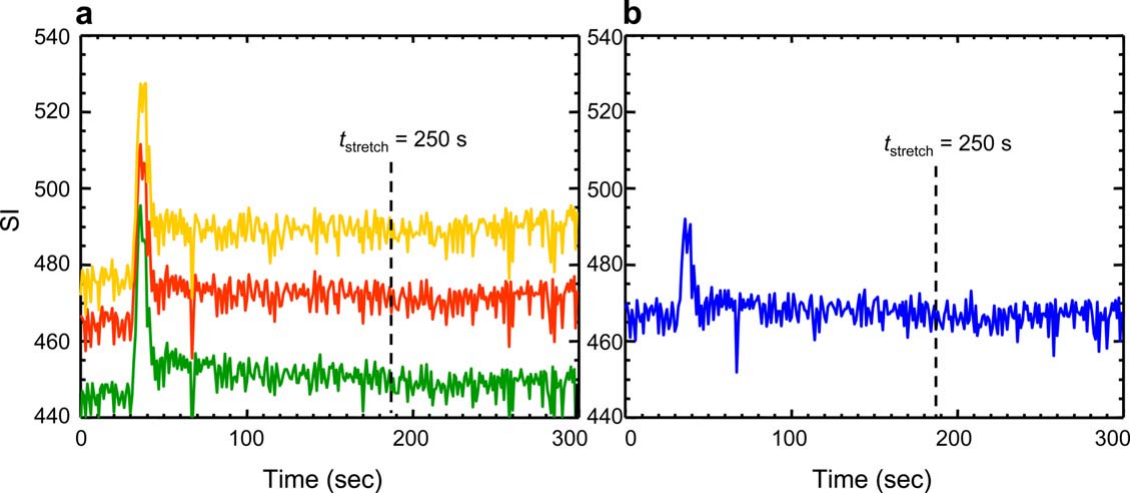
ROI‐averaged SI‐time curves showing dynamic enhancement in the four color‐coded regions, respectively, in white matter from the same patient as in Fig. 3. Each ROI was a collection of pixels coded with the same color in a WM‐segmented slice (slice 43, ie, the 1^st^ image in the middle row of Fig. 3).

ROI‐averaged SI‐time curves, which were obtained from the blue‐, green‐, red‐, and yellow‐coded regions, respectively, demonstrated variations in baseline level, peak height of first pass of CA bolus, and enhancement extent (Fig. 4).

### Quantitation of Tissue BBB Permeability With PP and the New Hybrid Method

**Figure 5:**
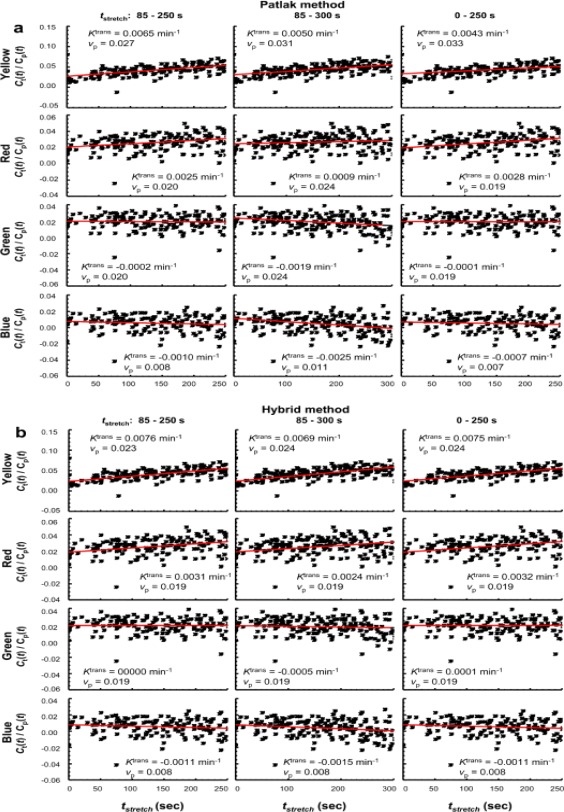
Fitting the tissue CA uptake curves from each of the four color‐coded regions. The Patlak analysis **(a)** and the hybrid method **(b)** were used, with three *t*_stretch_ intervals (left column: 85–250 sec; middle column: 85–300 sec; right column: 0–250 sec), respectively. The “measured” values of *K* ^trans^ and *v*_p_ are shown in the panel for each fitting.

Figure 5 shows the fittings of the four regional tissue CA uptake curves using the two kinetic approaches and three *t*_stretch_ intervals, respectively. For the *t*_stretch_ interval 85–250 seconds, the results from PP were quite close to those derived from the hybrid method, although the values of *v*_p_ estimated with PP were generally higher, and the values of *K* ^trans^ were generally lower, compared with their hybrid counterparts. Comparing results with various *t*_stretch_ intervals, the hybrid method showed better robust stability against variation in the *t*_stretch_ interval.

### Computer Simulation to Evaluate the New Hybrid Method

**Figure 6:**
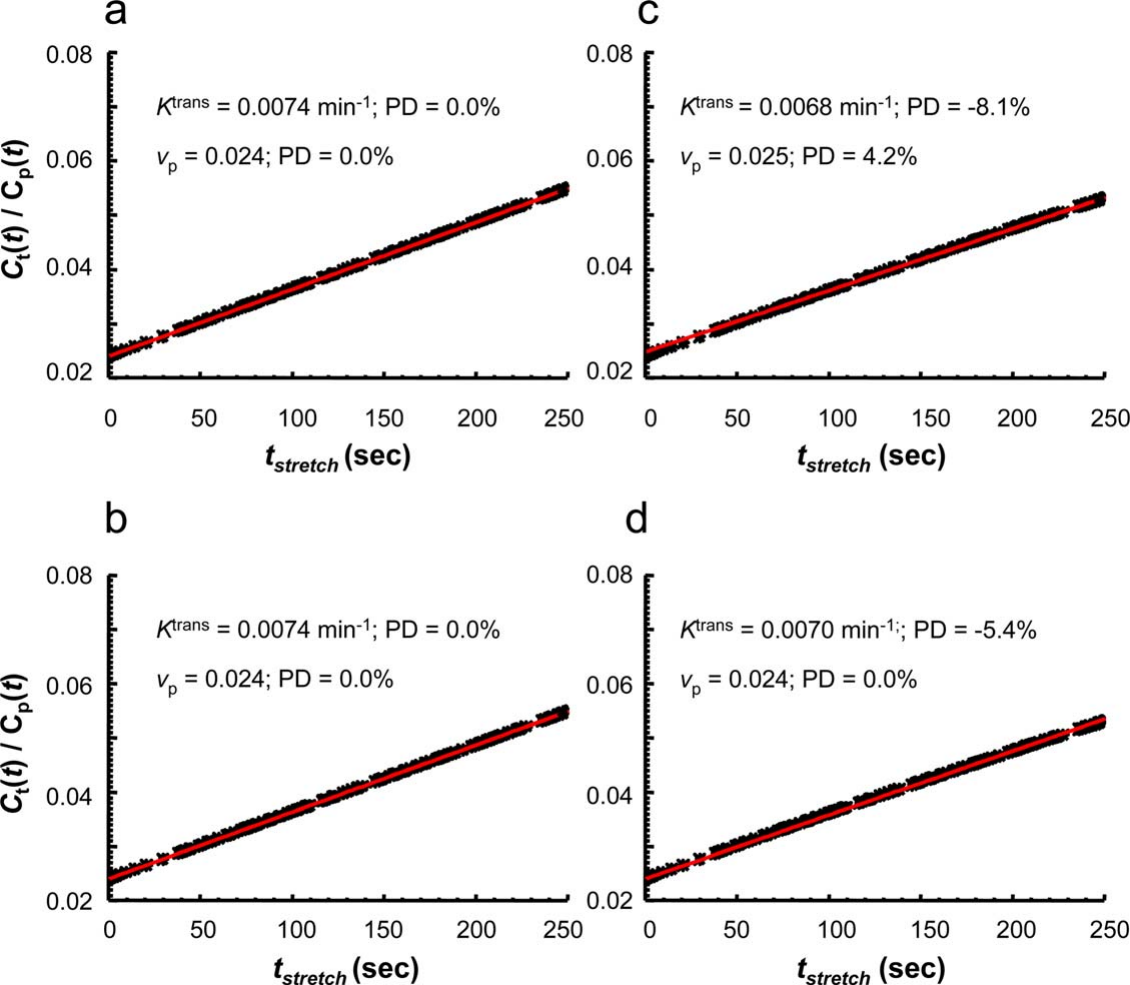
Zero noise tissue uptake curves synthesized with the unidirectional two‐compartment model **(a,b)** or the modified Tofts model **(c,d)**, which were fitted with the conventional Patlak analysis (a,c) or the new hybrid method (b,d) to assess the effects of ignoring backflux on the parameter estimates. These tissue uptake curves were simulated with “true” values of *K* ^trans^ = 0.0074 min^−1^ and *v*_p_ = 0.024. The panel in the upper‐left corner of each graph presents the “measured” values of *K* ^trans^ and *v*_p_ from the fitting. A *t*_stretch_ interval 85–250 seconds was used in the fitting.

Figure 6 shows that both PP and the hybrid model yielded parameter estimates equal to the true values when there is neither noise nor backflux (Fig. 6a,b). The Patlak analysis overestimates *v*_p_ (PD = 4.2%) and underestimates *K* ^trans^ (PD = –8.1%) when there is backflux (Fig. 6c). The impact of the backflux was reduced when the new hybrid method was employed (*v*_p_: PD = 0.0%; *K* ^trans^: PD = –5.4%) (Fig. 6d).

**Figure 7:**
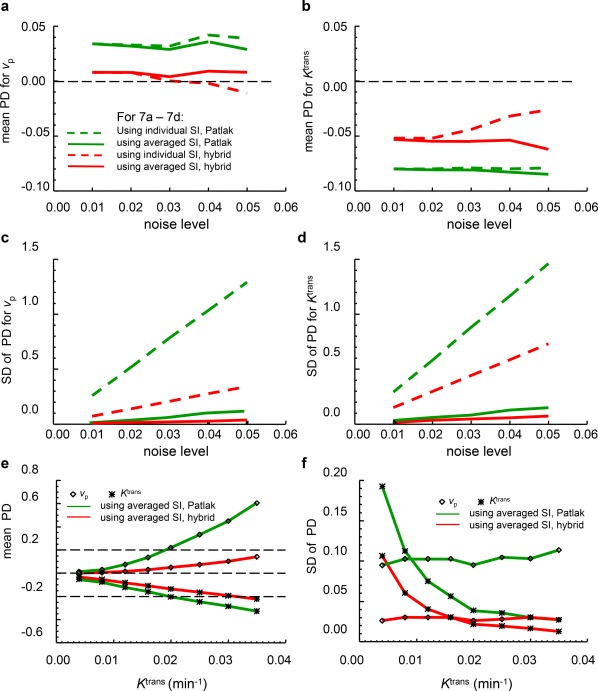
PD analysis for *K* ^trans^ and *v*_p_ estimates with varying noise levels **(a–d)**, or with various backdiffusion levels **(e,f)**. Mean (a,b) and SD (c,d) of percent deviations for *v*_p_ (a,c) and *K* ^trans^ (b,d) calculated from 20,000 Monte Carlo repetitions of fitting individual SI‐time curves (dashed lines) and 200 Monte Carlo repetitions of fitting an average of 100 individual SI‐time curves (solid lines) using the Patlak (green) and the hybrid (red) methods. The tissue uptake curves in a–d were simulated with “true” values of *K* ^trans^ = 0.0074 min^−1^, *v*_e_ = 0.20, and *v*_p_ = 0.024. In e,f, the tissue uptake curves were simulated with varying “true” *K* ^trans^ (0.004–0.035 min^−1^) but with a fixed noise level of 4%. A *t*_stretch_ interval 85–250 seconds was used in the fitting.

Figure 7a–d compares PD (mean and SD) in *v*_p_ and *K* ^trans^ derived from PP and the hybrid methods, at varying noise levels. The conventional Patlak analysis overestimates *v*_p_ and underestimates *K* ^trans^. The hybrid method estimates *v*_p_ accurately, and also reduced the bias in *K* ^trans^ estimation. With the new method, the SD of PD for both *v*_p_ and *K* ^trans^ estimates is greatly reduced. In addition, averaging the SI‐time curves simulated under the same conditions produces further improved the precision of both *v*_p_ and *K* ^trans^ for both methods. The simulation shows that, at a noise level of 4% (a noise level similar to that in the current in vivo study), it is theoretically possible to reach a PD of 0.9 ± 2.7% for *v*_p_, and a PD of –5.4 ± 5.9% for *K* ^trans^ using the new hybrid method combined with the SI averaging scheme. In comparison, the PP method could obtain a PD of only 3.6 ± 11.3% for *v*_p_, and a PD of –8.3 ± 12.8% for *K* ^trans^. These PD means are similar to the PD values shown in Fig. 6c,d. This means that, under simulation conditions, the bias (means of PD) is caused by the existence of backflux of CA, and the precision (standard deviations of PD) is dominated by the noise. The new hybrid method is shown to be more robust to effects from both the existence of backflux of CA and nonuniform (distorted) noise.

With a *t*_stretch_ interval 0–250 sec, the simulations showed remarkable improvement in the conventional Patlak analysis, but little effect on the hybrid method. That means that including the initial data points effectively reduced the effects of distorted errors on the Patlak analysis. The accuracy of both *K* ^trans^ and v_p_ estimates and precision of *K* ^trans^ were approaching those of their hybrid counterparts. The precision of v_p_, from Patlak, however, remained inferior to the estimate produced by the hybrid analysis. On the other hand, in vivo data fitting with a *t*_stretch_ interval 0–250 seconds (the right columns in Fig. 5a,b) showed good agreement between the two methods for uptake curves from the red‐, green‐, and blue‐coded regions; however, a strong overestimate of *v*_p_ and underestimate of *K* ^trans^ were seen for the yellow‐coded range using the Patlak method. This implies that some other factors, in addition to noise, affect the initial shape of the in vivo uptake curve, especially for the yellow‐coded region, and that the hybrid method is more robust in these conditions.

Figure 7e shows that, at a noise level of 4% with SI averaging, the conventional PP has started to yield *K* ^trans^ estimates with negative PD beyond –0.20 and *v*_p_ estimates with positive PD beyond 0.20 when *K* ^trans^ > 0.02 min^−1^ (*k*_ep_ > 0.1 min^−1^). In contrast, the new hybrid method started to yield *K* ^trans^ estimates with negative PD beyond –0.20 only when the “true” *K* ^trans^ > 0.030 min^−1^ (*k*_ep_ > 0.15 min^−1^), while PD mean for *v*_p_ estimates remains less than 0.20 even when *K* ^trans^ = 0.035 min^−1^. The SD of percent deviation for *K* ^trans^ estimates reduces when the “true” value of *K* ^trans^ increases, while the SD of PD for *v*_p_ estimates was not much influenced by the increase in the “true” value of *K* ^trans^.

### Correlation Between ΔR1 and *K* ^trans^ in WM

**Figure 8:**
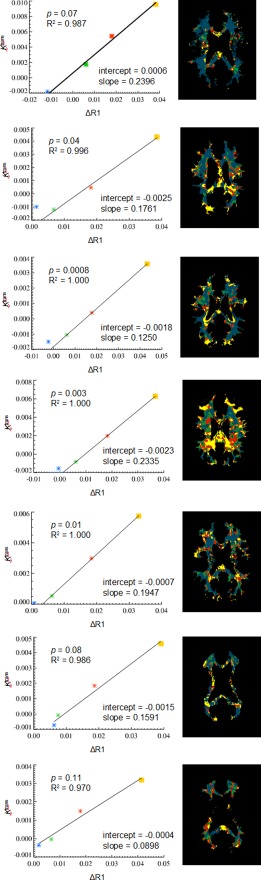
Linear regression analysis of the relationship between ΔR1 and *K* ^trans^ (left column) measured from the yellow‐, red‐, and green‐coded regions in WM segments in a representative slice from each of the seven patients (right column).

Figure 8 shows scatterplots of ΔR1 and *K* ^trans^ measured from the four color‐coded WM regions in a representative image slice for each of the seven patients. Linear regression analysis performed on the positive ΔR1 (yellow, red, and green) regions showed that ΔR1 and *K* ^trans^ are well correlated, with R^2^ = 0.991 ± 0.011 and *P* = 0.04 ± 0.04 (*n* = 7). The blue‐coded region contained voxels with negative ΔR1 and voxels with *v*_p_ less than 0.01 (their ΔR1 are generally low but may be positive); these were not included in the regression analysis. The correlation between ΔR1 and *K* ^trans^ indicated that WM‐segmented ΔR1 maps could be used to distinguish between regions in the WM with different *K* ^trans^ levels. However, the variation in the slope and the intercept of the linear regression among the seven patients (slope = 0.1740 ± 0.0548; intercept = –0.0012 ± 0.0011) implied that ΔR1 only could not determine the absolute value of *K* ^trans^.

### Comparison of the Four Color‐Coded Regions in WM

Table 1 illustrates multiple comparisons of mean values of ΔR1, *K* ^trans^, and *v*_p_ measured from four color‐coded WM regions in a representative image slice for each of the seven patients as presented in Fig. 8 (right column). One‐way ANOVAs showed significant variations from the four WM regions (*P* < 10^−15^ for ΔR1; *P* < 10^−6^ for *K* ^trans^; *P* < 10^−4^ for *v*_p_, respectively). Post‐hoc Tukey's HSD tests indicated statistical differences between the individual regions, except for the blue and green pair on *K* ^trans^ and the green and red pair on *v*_p_. Table 1 quantitatively revealed the regional inhomogeneity of BBB permeability in NAWM. It also revealed a trend of positive correlation between the *K* ^trans^ and *v*_p_ in NAWM.

**Table 1:** Means and Standard Deviations of ΔR1, *K* ^trans^, and *v*_p_ for Each Color‐Coded WM Region

	ΔR1				*K* ^trans^				*v*_p_			
Statistics (*N* = 7)	blue	green	red	yellow	blue	green	red	yellow	blue	green	red	yellow
mean	−0.000248	0.006370	0.018193	0.038593	−0.000887	−0.000225	0.002079	0.005324	0.007522	0.017173	0.018685	0.028903
sd	0.005839	0.000303	0.000311	0.003303	0.000767	0.001059	0.001728	0.002176	0.001177	0.001072	0.002373	0.012767
One‐way ANOVA	F = 180.0, *P* < 10^−15^				F = 23.4, *P* < 10^−6^				F = 12.5, *P* < 10^−4^			
Difference for each pair of regions (lower bound, estimate, upper bound), using Tukey's HSD test, Alpha = 0.05												
blue ‐ green	−0.0116 −0.0066 −0.0017*				−0.0029 −0.0007 0.0016				−0.0193 −0.0097 −0.0000*			
blue ‐ red	−0.0234 −0.0184 −0.0135*				−0.0052 −0.0030 −0.0007*				−0.0208 −0.0112 −0.0015*			
blue ‐ yellow	−0.0438 −0.0388 −0.0339*				−0.0085 −0.0062 −0.0039*				−0.0310 −0.0214 −0.0117*			
green ‐ red	−0.0168 −0.0118 −0.0069*				−0.0046 −0.0023 −0.0000*				−0.0112 −0.0015 0.0081			
green ‐ yellow	−0.0372 −0.0322 −0.0273*				−0.0078 −0.0055 −0.0033*				−0.0214 −0.0117 −0.0021*			
red ‐ yellow	−0.0254 −0.0204 −0.0154*				−0.0055 −0.0032 −0.0010*				−0.0199 −0.0102 −0.0006*			

Multiple comparisons of the regional means were performed using one‐way ANOVA followed by Tukey's all‐pairwise comparisons. The new hybrid method was used for deriving *K* ^trans^ and *v*_p_.

*The pair is statistically different.

### Robust Stability Against Variation in the *t*_stretch_ Interval

Table 2 lists the *K* ^trans^ and *v*_p_ derived from fitting the yellow‐ or the red‐coded regional curves as shown in Fig. 5a,b using four *t*_stretch_ intervals, respectively. Parameter mean from the four *t*_stretch_ intervals and corresponding CoV are also listed in Table 2 for the Patlak method, and for the hybrid method, respectively. The green‐ and blue‐coded regions were excluded from the CoV analysis due to existence of the negative *K* ^trans^ values in their fitting results. In agreement with the simulation results, in vivo data also showed the hybrid method superior to the Patlak in the robust stability against variation in the *t*_stretch_ interval.

**Table 2:** Stability Against Variation in the *t*_stretch_ Interval for the Patlak (**a**) and the Hybrid (**b**) Methods

	Yellow		Red	
	*K* ^trans^	*v*_p_	*K* ^trans^	*v*_p_
**Patlak**				
85‐250s	0.0065	0.027	0.0025	0.020
85‐300s	0.0050	0.031	0.0009	0.024
0‐250s	0.0043	0.033	0.0028	0.019
0‐460s	0.0045	0.032	0.0013	0.022
**Mean**	**0.0051**	**0.031**	**0.0019**	**0.021**
**CoV**	**19.6%**	**8.6%**	**49.0%**	**10.4%**
**Hybrid**				
85‐250s	0.0076	0.023	0.0031	0.019
85‐300s	0.0069	0.024	0.0024	0.019
0‐250s	0.0075	0.024	0.0032	0.019
0‐460s	0.0063	0.024	0.002	0.019
**mean**	**0.0071**	**0.024**	**0.0027**	**0.019**
**CoV**	**8.5%**	**2.1%**	**21.4%**	**0.0%**

## Discussion

Low‐level BBB permeability has been identified in apparently normal‐appearing white and gray matter in a range of diseases including cerebrospinal vessel disease, diabetes, dementia, stroke, multiple sclerosis, and systemic lupus erythematosis, as well as normal aging.^4^, ^17^, ^18^, ^19^, ^20^, ^21^, ^22^, ^23^, ^24^ Recent work has also identified changes in permeability in NAWM following whole brain radiotherapy in patients with cerebral tumors.^25^ The increase in BBB permeability induced by whole‐brain radiotherapy has been used to improve penetration of therapeutic agents for the treatment of primary central nervous system (CNS) lymphoma and metastatic disease typically depending on simultaneous measurement of cerebrospinal fluid (CSF) and plasma concentrations of the therapeutic agent.^26^, ^27^ In patients with glioblastoma and, to a lesser extent, some metastatic cerebral tumors, there is commonly extensive disease spread beyond the enhancing tumor areas identified by conventional imaging.^28^ There is therefore a need to effectively deliver drugs across the BBB, which has led to a growing interest in therapeutic manipulation of BBB permeability.^29^ The development of techniques for effective measurement of low levels of BBB permeability may provide a potentially valuable imaging biomarker both for prognostic/predictive purposes and for use in clinical trials where BBB permeability manipulation is planned.

We describe a new method, which combines the FP and PP approaches, to measure subtle CA leakage through the BBB using DCE‐MRI. Both the FP and PP approaches start from the same equation (Eq. [1]), but use different algorithms for deriving kinetic parameters. The FP approach produces more reliable *v*_p_ estimates using an integration technique, while the Patlak approach is superior for *K* ^trans^ estimation, collecting more data points beyond the first pass, which is necessary to measure subtle leakage. By combining the advantages of these two techniques, the hybrid method becomes more robust to effects from nonuniform (distorted) noise, variation in *t*_stretch_ interval choice, and error caused by the existence of backflux of CA.

A wide range of *t*_stretch_ intervals has been used in PP analysis in previous studies. For example, Larsson et al used intervals of 70–250 seconds and 140–250 seconds in a study with patients with brain tumors and healthy subjects,^2^ while Ewing et al performed the linear regression over a *t*_stretch_ interval of 30 minutes in their study with a rat model.^3^ In fact, heterogeneity in brain BBB permeability makes optimization of a *t*_stretch_ interval choice complicated. Shorter *t*_stretch_ intervals ensure that the linear regression is performed before major backflux of CA occurs and are appropriate for analysis of higher BBB permeability (yellow‐coded WM, eg). On the other hand, the longer *t*_stretch_ intervals should improve measurements of lower BBB permeability (red‐ and green‐coded WM, eg). A kinetic analysis method with the ability to minimize bias due to backflux and variation in *t*_stretch_ interval choice, such as we present here, will help in addressing this challenging problem.

This article also proposed the combined use of ΔR1 mapping and ROI‐based pharmacokinetic analysis for BBB permeability analysis in NAWM. The information on spatial distribution of residual CA within NAWM is useful since pixel‐by‐pixel kinetic analysis of WM tissues remains challenging due to a low signal‐to‐noise ratio (SNR) in the NAWM uptake curves. The color‐coded ΔR1 maps were used for automatic delineation of ROIs with different ΔR1 levels. The combined temporal‐ and spatial‐averaging scheme could minimize the effects of low SNR: frame‐averaged signal intensities were used to calculate R1_pre_ and R1_post_, producing ΔR1 maps, and then ROI‐averaged tissue uptake curves were used for kinetic analysis. In addition, the finding of a positive correlation between ΔR1 and *K* ^trans^ in NAWM shows that WM‐segmented ΔR1 maps may be used to demonstrate the spatial distribution of relative levels of BBB permeability.

The WM‐segmented ΔR1 maps and the pharmacokinetic analysis performed afterwards provided interesting information on the spatially inhomogeneous distribution of BBB permeability in NAWM. We found that a portion of WM voxels showed negative values of ΔR1 and *K* ^trans^, generally associated with low *v*_p_ values (<0.01). The mechanism for the apparent negative ΔR1 and *K* ^trans^ is not clear. The *K* ^trans^ and *v*_p_ values we observed in the yellow‐ or red‐coded WM regions were similar to those reported by Larsson et al^2^ and Heye et al.^17^ In addition, the WM regions with higher BBB permeability tend to have higher *v*_p_. These observations have the potential to provide quantitative information about WM microstructure and its changes in pathology.

Low dose (1/5 of the standard Gd‐DTPA dose) and high temporal resolution DCE‐MRI data were used in this study. Taheri et al used a quarter of the standard Gd‐DTPA dose and found that R1 in the vicinity of this reduced dose changed much more rapidly with changes in CA concentration than at higher concentrations.^6^ A lower dose of Gd‐DTPA entails less possibility of introducing a $T_{2}^{*}$ effect or truncation of the bolus peak of the arterial input function,^2^, ^30^ and also less risk of potential side effects of Gd‐DTPA in patients with impairment of renal function.^6^, ^31^ Our study supported that a dose as low as only 1/5–1/4 of the standard Gd‐DTPA dose was still adequate for performing the measurement of BBB permeability in WM. This would enhance the applicability of the technique.

Our study had some limitations. First, we evaluated the new hybrid method only in a small cohort of patients with NF2. Larger studies will be needed to apply the new method in a larger cohort, including patients and healthy subjects for comparison. Second, the plasma concentration curve we measured from the superior sagittal sinus has the merits of high SNR, distinctive peak of first pass, and is free of artifacts resulting from partial volume error or in‐flow signal loss.^32^ However, we realize that the ideal place to extract a vessel input function is from the arteries which supply the white matter. Nevertheless, to obtain a reliable measurement of *C*_p_(t) from the feeding arteries remains challenging.

In conclusion, this study proposed a new pharmacokinetic analysis method for the measurement of subtle BBB permeability in NAWM. Both computer simulation and in vivo study demonstrated improved reliability in *v*_p_ and *K* ^trans^ estimates. The combination of the pharmacokinetic analysis with pixel‐by‐pixel mapping CA‐induced *T*_1_ changes provides easy‐to‐apply and reliable imaging methods for evaluation of BBB permeability. The heterogeneous distribution of ΔR1, *v*_p_, and *K* ^trans^, and the relation between them revealed by MRI, may suggest differences in tissue composition between different locations in WM. How it relates to etiology and tissue destruction will be an interesting field to explore.

## Appendix A

By assuming that backflow during the first‐pass of the CA bolus was negligible, Eq. 1 can be used for kinetic analysis of FP MRI data.^7^, ^8^ Note that the two terms on the right side of Eq. [1] represent the intravascular (*C*_vas_(*t*)) and interstitial components (*C*_ees_(*t*)), respectively, ie:

(A.1) $$C_{vas}(t)\;=\;v_{p}C_{p}(t).$$

and

(A.2) $$C_{ees}(t)\;=\;K^{trans}\;\int_{0}^{t}C_{p}(\tau )d\tau .$$

Eq. [A.2] indicates that the interstitial component of the bulk tissue concentration, C_ees_(t), is proportional to the integral of the input curve when backflux of contrast is not included in the model. Separation of the intra‐ and extra‐components of the enhancement curve during the first pass of the CA bolus was based on the integral of the input curve, which was termed the “leakage profile” (LP) in previous studies^7^, ^8^:

(A.3) $$LP(t)=\int_{0}^{t}C_{p}(\tau )\;\;d\tau ,\;\;\;\;0\leq t\leq T_{rR},$$

where *T*_rR_ is the time of the beginning of the recirculation phase identified from the input function.^7^, ^8^

An iterative analysis was proposed for separation of the intra‐ and extra‐components of the enhancement curve^8^:

(A.4) $$C_{vas}(t)\;=\;C_{t}(t)-K^{trans}\cdot LP(t).$$

The FP model calculates the corrected relative cerebral blood volume with an integration method, a similar strategy to the technique described in dynamic susceptibility‐contrast enhanced (DSC) MRI^33^, ^34^, ^35^:

(A.5) $$rCBV_{correctd}^{T1}\;=\;\;\;\int_{0}^{t=T_{rR}}C_{vas}(t)dt,$$

and

(A.6) $$v_{p}=\int_{0}^{t=T_{rR}}C_{vas}(t)dt/\int_{0}^{t=T_{rR}}C_{p}(t)dt.\;\;\;\;\;\;\;\;\;\;\;$$

## Appendix B

The 4D LDHT DCE‐MRI data were spatially aligned with and resliced to the high spatial resolution series of the ICR‐DICE,^11^, ^30^ using SPM2.^13^ The voxel size of the spatially aligned and resliced HT images is much smaller, ie, 1 × 1 × 2 mm^3^ (after reslicing) vs. 2.5 × 2.5 × 6.35 mm^3^ (before reslicing). With the high temporal resolution (Δ*t* = 1s), for catching up the peak of Gd‐contrast enhancement, the SSS could be easily outlined in several consecutive slices of the axial orientation image slab, in spite of the low contrast dose.

A semiautomatic procedure for *C*_p_(*t*) extraction was used. Operator interaction is limited to the identification of: 1) The axial slab for searching SSS ROIs. The operator should first identify the time frame that caught the peak of the first pass of CA bolus in the SSS, and then find the axial slab (in the 3D volume identified) over which the SSS spanned across. The two end slices of the predefined axial slab were denoted as SL_i‐end_ and SL_i+end_. 2) The slice where the automatic searching started (SL_i_). The starting slice was chosen, whereas the SSS was approximately perpendicular to the image plane, preferred to be near the center slice of the predefined axial slab. A small oval or rectangle ROI of a × b was manually drawn on the selected slice (SL_i_), just covering the SSS. The ROI associated with the selected slice (SL_i_) was denoted as ROI_i_.

The automatic searching for SSS ROIs was performed in the order of SL_i‐1_, SL_i‐2_, …, SL_i‐end_, and then SL_i+1_, SL_i+2_, …, SL_i+end_. These ROIs were predefined as of the same shape and size as ROI_i_, but might need to be slightly repositioned along the SSS runs. The searching on slice SL_i‐1_ started from the X‐Y‐coordinates coincided with the center of ROI_i_, but within a region with extended size (2a × 2b). The search ended when the ROI_i‐1_ achieved its maximum mean SI values. The search of ROI _i‐2_ on SL _i‐2_ repeated as for ROI_i‐1_, but in a 2a × 2b region whose center coinciding with the X‐Y‐coordinates of the center of ROI_i‐1_, and so on. As such, candidate voxels were collected along the SSS over the whole imaging volume. The SI time course for each of the collected voxels was extracted from the 4D LDHT. The area under the SI enhancing curve within 30 seconds of the arrival time (AUC_30_) was calculated. A mean SI(*t*) curve was calculated from *N* (˜50) voxels, which had the highest AUC_30_. The averaged mean SI(*t*) was then converted to the plasma CA concentration time course curve (*C*_p_(*t*)). The number of voxels, *N*, for the calculation of the final *C*_p_(*t*), can be altered as different data acquisition protocols are used. In principle, the finer of the spatial resolution, the more voxels could be used for the calculation.

Bolus arrival time (BAT) was estimated for each tissue uptake curve. The C_p_(*t*) measured from the SSS has to be time‐shifted to match the BAT for the kinetic analysis.

## Appendix C

The theoretic prediction of the steady‐state signal, *S*, from a transverse‐spoiled gradient‐echo acquisition, assuming short echo time (TE ≪ $T_{2}^{*}$, $T_{2}^{*}$ is the effective transverse relaxation time), is given by:

(C.1) $$S=M_{0}\cdot \sin\alpha \cdot \frac{1-\exp(-\text{TR}\cdot R1)}{1-\cos\alpha \cdot \exp(-\text{TR}\cdot R1)},$$

where *α* is the flip angle, TR is the repetition time, R1 is the longitudinal relaxation rate, and M_0_ is the equilibrium longitudinal magnetization, which depends on the proton density, the profile of the receiver coil sensitivity, the receiver gain setting, and the scaling parameters used for image reconstruction.^36^

Maps of M_0_ and R1_N_ were calculated by fitting the VFA signals with Eq. [C.1]. A nonlinear least squares method was used in the calculation of 3D R1_N_ and M_0_ maps.^7^, ^12^

## Appendix D

There are two ways for calculation of R1_post_ based on R1_N_, mean preinjection signal intensity (SI_pre_), and postinjection signal intensity (SI_post_):

By division:

(D.1) $$SI_{post}/SI_{pre}=\frac{1-\exp(-\text{TR}\cdot R1_{post})}{1-\cos\alpha \cdot \exp(-\text{TR}\cdot R1_{post})}\cdot \frac{1-\cos\alpha \cdot \exp(-\text{TR}\cdot R1_{N})}{1-\exp(-\text{TR}\cdot R1_{N})}.$$

By subtraction^16^:

(D.2) $$SI_{post}-SI_{pre}=M_{0}\cdot \sin\alpha \cdot (\frac{1-\exp(-\text{TR}\cdot R1_{post})}{1-\cos\alpha \cdot \exp(-\text{TR}\cdot R1_{post})}-\frac{1-\exp(-\text{TR}\cdot R1_{N})}{1-\cos\alpha \cdot \exp(-\text{TR}\cdot R1_{N})}).$$

The subtraction method needs both R1_N_ and M_0_ for calculation of R1_post_. The M_0_ value obtained from fitting of the VFA signal can be used for this purpose only when all the factors affecting the value of M_0_ are identical in the VFA and DCE sequences. That can be achieved when the DCE acquisition repeats one of the sequences in the VFA acquisition. In this study, the subtraction method was used for R1_post_ calculation to reduce computation instability that might occur in case of fluctuation in small values of SI_pre_.^16^, ^37^
